# An Interesting Case of Hysteria

**Published:** 1894-12

**Authors:** James Stafford

**Affiliations:** Gynecologist to the West Side German Clinic; New York Clinic of Specialties, etc.; 142 West 70th Street


### Clinical Report.

### AN INTERESTING CASE OF HYSTERIA WITH A TEMPERATURE OF 105.8°.

By JAMES STAFFORD, M. D.,
Gynecologist to the West Side German Clinic; New York Clinic of Specialties, etc.

Last March a case was referred to me by Dr. C. P. Biggs of this city, and as it was of unusual interest, I take the liberty of reporting it, for your consideration. A young girl of 19 years, single, whose early life, up to within a year previous, had been spent in a "convent school," was the patient.

She had always been in perfect health up to about five months preceding this illness, when she had acceded to the solicitations of a young man, under the promise of marriage, and became ènceinte. The young man refused to marry her, and to avoid disgrace, a premature delivery had been brought on, after which the girl had been well, except for the fact that she was depressed by the young man's refusal of marriage, and the consideration of the idea that she was no longer virtuous.

When I first saw the case, she had been having hysterical seizures each night, often accompanied by vomiting, crying, moaning, etc., so that the doctor had been called nearly every night for the past week. At one or two times he had found her temperature 102.5° F., by mouth. She has been kept in bed upon a light diet of beef juice and milk, and during the attacks, morph. sulph., grain one-fourth, had been administered by needle. This, together with moral suasion, composed about all the treatment used.

I was introduced to the patient in the afternoon, and, the next night, was hurriedly called to find her in contortions, of a purely hysterical character. I gave her a hypodermic of morph. sulph., grain one-fourth, and half a drachm each of fluid extract viburnum prunifolium, and fluid extract valerian every two hours when awake. I called the next day, finding the patient more easy, and requested that she sit up in a chair during the afternoon, having previously made a careful chest examination, and having found the heart and lungs in normal condition. The same treatment was continued. The following day she was sitting up and looking well, but, to my surprise, as I placed my hand upon her wrist, it was extremely warm, and the pulse was beating 125 per minute. Her temperature was 104.5°. I ordered her to bed and gave acetanalid, grains 5, every two hours with cold spongings and tincture aconite M. 2, every hour for three doses, and in four hours the temperature and pulse were normal. The next day she was improved, and I advised that she take a drive through the park, which was done. The following day, when I called, she was sitting in the parlor, and said she felt well and was desirous of attending the theatre that evening, if I would permit her to do so. To my surprise, upon taking the temperature by mouth, I found it 105.8°, and the pulse 140 per minute. I took the patient in my arms, carried her to the bed, and again examined her carefully, but could find no other cause for the temperature. I then took her to the bath-tub, laid her down in it and turned on lukewarm water, gradually cooling the water to about 80°, leaving her in the tub about ten minutes, and until the temperature had lowered about two degrees and she had begun to tremble with the cold and to beg me to let her out of the tub.

The next day she was feeling fine, and the temperature was normal. I told her she might go to the theatre, driving or anywhere else, but she must take a bath as cold as she could tolerate each morning. The attacks never returned, and for the past three months she has been perfectly well. The chief point of interest about the case I regard as the exaggerated heat production. Many cases of hysteria are seen by every physician, but one with such a temperature I regard as uncommon, and when such a high temperature is found, it is often apt to startle the physician and cause him to be somewhat skeptical about his abilities as a diagnostician.

142 West 70th Street.

